# A Time-, Gender-, and Disease-State Invariant Model of Fitness Across the Adult Lifespan

**DOI:** 10.1093/geroni/igaa057.1287

**Published:** 2020-12-16

**Authors:** Scott Maitland, Paula Brauer, David Mutch, Dawna Royall, Doug Klein, Angelo Tremblay, Caroline Rhéaume, Khursheed Jeejeebhoy

**Affiliations:** 1 University of Guelph, Guelph, Ontario, Canada; 2 University of Alberta, Edmonton, Alberta, Canada; 3 Laval University, Québec, Quebec, Canada; 4 University of Toronto, Toronto, Ontario, Canada

## Abstract

In studies of community-based health behavior interventions (diet and physical activity) one goal in analysis is to show expected relationships between measures of intervention and clinically relevant outcomes. Many programs fail to show such clear links for many reasons beyond lack of intervention effectiveness. These secondary analyses were undertaken to assess if the measurement properties (stability and responsiveness) of intervention measures could have contributed to study findings. A feasibility study of lifestyle treatment of metabolic syndrome (n=293; mean age = 59yrs) had achieved 19% reversal over one year, yet neither diet quality nor fitness were associated with cardiovascular disease risk. Confirmatory factor analysis was used to examine fit of measurement models and factorial invariance was tested across three time points (baseline, 3-month, 12-month), gender (male/female), and disease status (diabetes) for the Healthy Eating Index (HEI) (Canada 2005) and several fitness measures (VO2max, flexibility, curl-ups, push-ups). The model fit for HEI was poor and could account for the lack of association seen in the original study. More development of diet quality measures is needed. The model for fitness, however, demonstrated excellent fit and displayed measurement equivalence across time, gender, and disease state. A higher degree of confidence exists when measurement equivalence/invariance is demonstrated, allowing for reliable tests of differences in comparison groups. The use of a multiple measure of fitness, including cardiorespiratory fitness, flexibility, and strength, helps eliminate limitations of using measures from a single domain or self-reported data is promising and should be considered in future work.

